# Usability and usefulness of (electronic) patient identification systems—A cross-sectional evaluation in German-speaking radiation oncology departments

**DOI:** 10.1007/s00066-023-02148-9

**Published:** 2023-09-15

**Authors:** Andrea Baehr, Maximilian Grohmann, Maja Guberina, Katrin Schulze, Tim Lange, Ursula Nestle, Philipp Ernst, DEGRO Working Group for Patient Safety

**Affiliations:** 1 Department of Radiation Oncology, University Hospital Hamburg-Eppendorf, Hamburg, Germany; 2 Department of Radiotherapy, University Hospital Essen, West German Cancer Center, University Duisburg-Essen, Essen, Germany; 3 Department of Radiation Oncology, Fulda Community Hospital, Fulda, Germany; 4 Clinic for Radiotherapy, Hannover, Medical School, Hannover, Germany; 5 Department of Radiation Oncology, Kliniken Maria Hilf GmbH, Moenchengladbach, Germany; 6 Department of Radiation Oncology, University Hospital Hamburg-Eppendorf, Martinistr. 52, 20246 Hamburg, Germany

**Keywords:** Patient safety, Adverse event, Incident, Safety barrier, Biometric methods

## Abstract

**Purpose:**

Patient misidentification in radiation oncology (RO) is a significant concern due to the potential harm to patient health and the burden on healthcare systems. Electronic patient identification systems (ePIS) are increasingly being used as an alternative or supplement to organizational systems (oPIS). The objective of this study was to assess the usability and usefulness of ePIS and oPIS in German-speaking countries.

**Methods:**

A cross-sectional survey was designed by a group of experts from various professional backgrounds in RO. The survey consisted of 38 questions encompassing quantitative and qualitative data on usability, user experience, and usefulness of PIS. It was available between August and October 2022.

**Results:**

Of 118 eligible participants, 37% had implemented some kind of ePIS. Overall, 22% of participants who use an oPIS vs. 10% of participants who use an ePIS reported adverse events in terms of patients’ misidentification in the past 5 years. Frequent or very frequent drop-outs of electronic systems were reported by 31% of ePIS users. Users of ePIS significantly more often affirmed a positive cost–benefit ratio of ePIS as well as an improvement of workflow, whereas users of oPIS more frequently apprehended a decrease in staffs’ attention through ePIS. The response rate was 8%.

**Conclusion:**

The implementation of ePIS can contribute to efficient PI and improved processes. Apprehensions by oPIS users and assessments of ePIS users differ significantly in aspects of the perceived usefulness of ePIS. However, technical problems need to be addressed to ensure the reliability of ePIS. Further research is needed to assess the impact of different PIS on patient safety in RO.

**Supplementary Information:**

The online version of this article (10.1007/s00066-023-02148-9) contains supplementary material, which is available to authorized users.

## Introduction

Errors in patient identification (PI) can lead to serious harm to the patients’ health and can affect the economics of health sectors [1, 2]. Providing a reliable method for PI is especially important in radiation oncology (RO), where severely ill patients are treated on multiple consecutive days and the staff faces a high number of treatments per day [3, 4]. Several consensus documents recommend daily procedures to guarantee correct PI in RO and healthcare in general [5–7]. Nevertheless, patient misidentification remains a significant issue in radiology departments around the world [3, 8–11]. Recently, the German Federal Bureau for Radiation Protection published their report on serious incidents in 2021, stating patient misidentification as the main reason for reportable events in Germany, accounting for 34% of the reports (18/51 events; [12]).

The US Joint Commission declared patient identification as the top priority for the health sector in 2023 [13], underlining comparable to World Health Organization (WHO) statements that PI should rely on at least two different methods [14]. The PI methods often enclose organizational patient identification systems (oPIS) such as asking for patient-specific identifiers (e.g., date of birth). Although oPIS are highly effective for most cases [1], several team-related factors, patient factors, and problems with equipment can contribute to failures [3]. Currently, many oPIS are being replaced or supplemented by electronic systems (ePIS) that use biometric methods (such as face recognition) or digital systems that utilize barcodes or RFID (radiofrequency identification) technology [15].

While biometric methods offer advantages such as very high sensitivity (fingerprints 65–100%, facial recognition 99.7% [16]), limitations such as vulnerability to changes in biometric characteristics [17] can affect their reliability. In addition, there are also ethical and legal concerns regarding data privacy [18] that need to be considered. Despite being in use for several years in RO, there are only a few published accounts of the performance of different ePIS in this setting. For example, Wegner et al. showed that utilizing an electronic identification and patient organization system can enhance processes, resulting in higher satisfaction among patients and staff [19]. To date, no publication offers an evaluation of the accuracy of oPIS explicitly in RO. Overall, we face a lack of data concerning different aspects of merit such as usability for both oPIS and ePIS.

*Usability* is defined as the extent to which a certain product or process can be used by specific users to achieve goals with effectiveness (accuracy and completeness), efficiency (resources expended in relation to effectiveness), and user satisfaction (comfort and acceptability of use; [20]). *Perceived usefulness* is a main part of technology acceptance and is defined as the degree to which a person believes that using a particular system would enhance his or her job performance [21]. Measuring usability and perceived usefulness of healthcare systems using different questionnaires is well described in the literature [21–23].

The aim of our investigation was to evaluate the usability and perceived usefulness of ePIS and oPIS in German-speaking countries. To gather this information, we designed a cross-sectional survey that included a range of qualitative and quantitative questions about experiences with and expectations for ePIS and oPIS.

## Materials and methods

The questionnaire was created by experts from various professional backgrounds including physicians, medical physicists, radiation therapists, nurses, and administration staff during multiple conferences organized by the Working Group on Patient Safety of the German Society for Radiation Oncology (DEGRO). A core group of one radiation therapist, five physicians, and two physicists finalized the questionnaire and prepared the data and manuscript. The survey included a total of 38 questions in the five sections “general information,” “oPIS,” “ePIS,” “problems with identification of patients,” which mainly asked for qualitative data, and “experiences with PIS,” where participants should agree to different quotes about PIS. The latter was ranked with a 5-point Likert-scale (−2=*strongly disagree*, −1=*disagree*, 0=*neutral*, 1=*agree*, 2=*strongly agree*).

The survey was online available from August to October 2022. Invitations were sent via the DEGRO mailing list, which includes 1434 members in German-speaking countries. One answer per professional group per department was requested. All answers remained anonymous, and all participants gave informed consent for publication. The data were analyzed using SPSS Statistics 28 (IBM, Indianapolis, IN, USA). The Man–Whitney *U* test was used to compare median values of different groups and categorial data were compared via the chi-square test.

## Results

### Study population and PIS procedures

A total of 118 questionnaire forms were eligible for evaluation. Of those, 33 (28%) were participants from university hospitals, 39 (33%) from other hospitals, and 44 (37.3%) from medical practices, mainly in Germany, as well as Switzerland (*n* = 1) and Austria (*n* = 4). Most participants reported treating 1000–2500 patients per year (45%). Of all participants, the majority were physicians (58%) or medical physicists (23%), 13% were radiotherapists, and 7% were nurses or administrative staff.

A total of 116 participants (98%) reported having a concept for PI, of which 99 (85%) have a written standard operating procedure. Among the 51 participants who use an ePIS, 49% (*n* = 25) have used it for more than 3 years, 20% (*n* = 10) for 1–3 years, 10% (*n* = 5) for less than 1 year, and 11 did not respond to this question.

Table 1 presents the different types of PIS. Further specifications of organizational and electronic procedures and systems are indicated in Table 2. A total of 51 departments have implemented some kind of ePIS. The number of patients per year did not show an impact on the existence of ePIS. However, hospitals other than university hospitals showed a trend toward a higher implementation rate of ePIS (51%) vs. university hospitals (39%) or medical practices (36%).

**Table 1:** Patient identification systems as reported in the survey responses (*n* = 118)

Patient identification systems (PIS)	*N*	%
Organizational procedures (e.g., call, portrait photograph)	65	55
Electronic manually procedures (e.g., barcode scanner and wristband)	21	18
Electronic automatic procedures (e.g., face recognition)	9	8
Combination of oPIS and ePIS	21	18
No answer	2	2

*oPIS* organizational patient identification system, *ePIS* electronic patient identification system

**Table 2:** Organizational and electronic patient identification (PI) procedures and products used for electronic PI. Multiple answers possible. Total number of participants who use organizational procedures = 65, total number of participants who use electronic procedures = 51

Organizational procedure for PI	Number of nominations (*N* = 86)	%
Calling the patient in the waiting room	73	85
Patient confirms identity when entering the treatment room	43	50
Patient states their own name when entering the treatment room	43	50
Patient states date of birth	31	36
Patient states address	5	6
Comparing patient’s portrait photograph	73	85
Comparing patient’s passport/ID card	13	66
Electronic procedure for PI	(*N* = 51)	–
Wristband with code	22	43
ID card	11	22
Fingerprint scanner	2	4
Face recognition	8	16
Other	4	8
No answer	10	20
Manufacturers/products for electronic PI	(*N* = 51)	–
Opasca	8	16
CRAD	5	10
VisionRT	5	10
Elekta	9	18
Siemens	1	2
MediloX	1	2
Wolf und Appenzeller	1	2
Varian or Mosaiq	9	22
Other	1	2
No answer	15	29

*PI* patient identification

### Effectiveness of PIS

Of the 65 participants who use an oPIS, 14 (22%) reported serious reportable events (according to the national radiation protection act) related to PI, involving up to five incidents in the past 5 years. Misidentification of patients upon their entry to the treatment room was cited as the main cause. Out of 51 participants who use an ePIS, five (10%, difference not significant) reported experiencing up to five events in the past 5 years. Departments with > 1000 patients per year reported more often about serious events than smaller departments did (32% vs. 15%, not significant).

Among 65 participants who use an oPIS, 51% (*n* = 33) reported having experienced one or more near-events related to patient identification in the past 5 years, compared to 28% (*n* = 18) of 51 participants who use an ePIS in addition to an oPIS. This difference was not statistically significant. Altogether, 16 of 65 (25%) participants using an oPIS only nominated the identification by the radiotherapists to be main cause of near-events, as well as to be the main barrier for misidentification (*n* = 13, 20%).

Of all departments with oPIS, 69 (70%) perform the identification process with every fraction, others reported performing it for the first fraction or change of staff only (*n* = 9, 10%) or during a certain number of fractions until the team knows the patient well (*n* = 8, 9%). With an ePIS, 36 of 51 departments use the system before each fraction (71%), while 13 participants (25%) did not answer this question.

### Efficiency of PIS

Of 65 participants who use only organizational procedures to identify patients, 50 (77%) use a combination of at least three different PIS, with an average of four methods being used. Most commonly, a call for the waiting patients (*n* = 58, 89%), active citation of their names (*n* = 36, 55%), and comparison with a photograph (*n* = 58, 89%) were nominated (compare Table 2). Departments with a combination of oPIS and ePIS (*n* = 21) reported a mean number of three organizational methods. A call for the waiting patients (*n* = 14, 67%) and comparing a photograph (*n* = 15, 71%) were still the most common method. The patient’s photograph also was the most common tool for identifying a patient unable to answer a call or question (21%, *n* = 18).

Of 51 participants using ePIS, 24 reported on the performance of their systems. Eight reported about weekly–daily and eight about quarterly–monthly deficiencies of the system (33% each). Of these, 12 (50%) reported about quarterly–monthly and four (17%) about weekly dropouts with their inability to identify the patient. In these cases, patients are commonly identified by one or two staff members who state their name or date of birth (*n* = 23, 45%).

### User satisfaction of ePIS

A total of 30 of 51 (59%) participants recommend their electronic device for identification. Of all participants who use manual ePIS (e.g., with a barcode), 62% recommend it vs. 77% of participants using automatic ePIS (e.g., face recognition) vs. 48% using a combination of oPIS and ePIS; 18% did not answer this question.

### Expected and perceived usefulness of ePIS

**Fig. 1:**
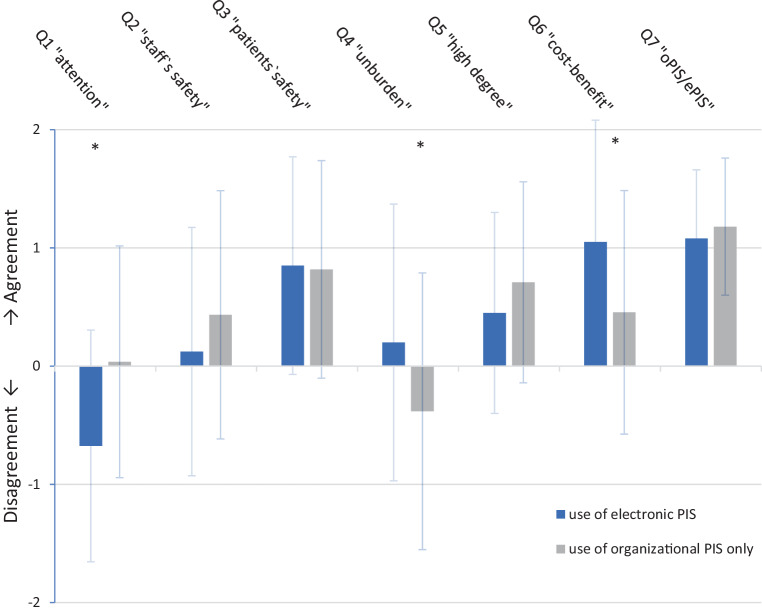
Mean agreement plus standard deviation levels of participants who use any kind of electronic patient identification system (*ePIS*, *light blue*) and participants who use an organizational patient identification system (*oPIS*, *dark blue*) only to the quotes (*Q*) 1–7 (see Table 3). (Scale: −2=*strongly disagree*, −1=*disagree*, 0=*neutral*, 1=*agree*, 2=*strongly agree*; * significance)

Among 65 departments with oPIS, 54 (83%) considered implementation of an ePIS. Of these, 49 (90%) nominated “increase of security” as the main argument for implementation; “confirmability for patients” and “simplification of processes” were nominated by 20 participants (37%) each. “High costs” (*n* = 22, 41%) and “high effort for implementation” (*n* = 20, 37%) were considered as main arguments contra ePIS, whereas an expected “limited functionality” was nominated by 10 participants (19%). Of all participants reporting on events or near-events in their departments in the past 5 years, 62% think that an electronic system could have prohibited these. The agreement to different quotes concerning use of ePIS is represented in Fig. 1 and Table 3.

**Table 3:** Statements of participants using oPIS and ePIS concerning quotes (Q) 1–7. Participants ranked the quotes from *strongly disagree* (−2) to *strongly agree* (2)

Quote	Group	Agreement/strong agreement (*n*, %)	Neutral (*n*, %)	Disagreement/strong disagreement (*n*, %)	No answer (*n*)	Mean value for agreement	*p* (mean value for agreement)
Q1	oPIS	18 (28)	24 (37)	13 (20)	8	0.04	< 0.01
	ePIS	5 (10)	11 (22)	24 (47)	13	−0.68	
Q2	oPIS	29 (45)	15 (23)	11 (17)	8	0.13	0.2
	ePIS	25 (49)	8 (12)	13 (20)	13	0.44	
Q3	oPIS	37 (57)	14 (22)	4 (6)	8	0.85	0.9
	ePIS	31 (61)	6 (12)	3 (6)	13	0.88	
Q4	oPIS	14 (22)	15 (23)	28 (43)	8	−0.38	0.015
	ePIS	15 (29)	14 (27)	9 (18)	13	0.2	
Q5	oPIS	43 (66)	15 (23)	4 (6)	8	0.71	0.25
	ePIS	22 (43)	9 (18)	9 (18)	13	0.45	
Q6	oPIS	29 (45)	16 (25)	10 (15)	8	0.45	0.03
	ePIS	34 (67)	5 (10)	1 (2)	13	1.1	
Q7	oPIS	52 (80)	3 (5)	0	8	1.1	0.3
	ePIS	34 (67)	6 (12)	0	13	1.2	

*Q1* “The staff’s attention decreases with use of an electronic identification system.” *Q2* “Staff feels safer with use of an organizational system.” *Q3* “Patients feel safer with the use of an electronic system.” *Q4* “An electronic system unburdens the staff and enables the team to focus on other tasks.” *Q5* “An organizational system for patient identification offers a high degree of safety.” *Q6* “The increase in safety legitimates costs and effort of implementation of an ePIS (cost–benefit ratio).” *Q7* “An ePIS should be accomplished by organizational measures.”

## Discussion

Radiation oncology departments are familiar with easy-to-apply identification protocols [24], which were ranked as a top efficient mechanism to enhance patient safety in the 2017 OECD report [1]. Nevertheless, the processes of RO are exposed to increasing patient numbers per treatment unit as well as permanent stressors and disruptions [25, 26], making them prone to errors that restrict the effectivity of the systems [27, 28]. Congruent with the finding of Hendee and Herman, who favor electronic safety barriers over procedures [29], an increasing number of departments implement ePIS to compensate for human liabilities, and accordingly, 43% of participants in our survey have implemented an ePIS in their departments.

Our participants expressed several expectations and concerns regarding the implementation of ePIS. The majority expected an increased security with the use of ePIS. In fact, 10% of ePIS and 22% of oPIS users reported on serious events in their departments in the past 5 years. Even though this difference was not significant, it indicates the chance to increase the effectivity by using or supplementing ePIS, as similarly described in other studies [30]. As with the use of ePIS the incidence of errors is still high, it is of immense interest to evaluate reasons for the system failures and methods to improve any kind of PIS in their effectivity.

Reason for failures of oPIS are mainly protocol violations, e.g., due to time pressure [31], or omission of procedural PIS for some treatment days [32]. In our investigation, only 70% of our participants use the oPIS protocol for each treatment day, whereas others only follow the protocol for the first treatment or change of treatment unit. Failures of ePIS can equally be connected to omission of use, e.g., in stressful situations [32], or with decreasing attention for any reason. Interestingly, a significantly higher proportion of participants with oPIS feared such a decrease in attention compared to the rating of those who in fact use ePIS. All-automatous ePIS with verification of patients’ identity before every fraction might preclude omission of the protocol, yet automation bias, meaning a too-strict belief in the accuracy of electronic systems, bares an important risk in all-automatous settings [33]. Data showed positive effects of use of team-time-out in the RO setting to overcome protocol omissions and might be implemented to increase effectivity of any PIS [34, 35].

The efficiency of ePIS might be inhibited by system drop-outs with the need for other patient identifiers to continue. In fact, 12 participants reported on a high and four on a very high frequency of system dropouts. The supplementation of oPIS to any implemented ePIS was supported by the great majority. It is noteworthy that on average, participants who used both ePIS and oPIS typically used a combination of three oPIS and one ePIS. It seems logical that the efficiency can be further increased with the reduction to, e.g., one ePIS and one oPIS in combination, which fulfills the WHO recommendation [14].

Most participants using oPIS do not expect a facilitation for the staff by ePIS, whereas, by contrast, participants using ePIS showed a significant agreement with the respective quote. This aspect indicates a possible increase in user satisfaction through use of ePIS. Data from the literature adumbrates a facilitation of the patient paths in RO through the use of an RFID-based system in RO [19], which might contribute to the easing of processes for the staff. Both groups believed that staff feel safer when using oPIS, while the majority of participants in both groups agreed that ePIS could enhance the perception of patient safety. Indeed, emerging evidence suggest that the role of the patient in ensuring patient safety is relatively underestimated, and there is potential for the development of a more robust safety culture [3, 36].

High costs for implementation were named as a main argument against ePIS. Despite this, especially those who already use ePIS in our investigation agreed that the increase in safety legitimates the high costs and efforts. In fact, digital solutions for safety issues were ranked as the most efficient for improving patient safety [1], considering that the economic burden of adverse events for public hospitals was estimated to be up to 32% of their budget in developed countries.

### Limitations

Our investigation has several limitations, including a low response rate, an unequal distribution of professions among participants, a limited number of participants, and a small representation of each individual ePIS system. In addition, there could be bias as departments without clear concepts for PI may have been hesitant to answer our survey. Nevertheless, our results offer an important insight into the perceived usability and usefulness among RO professionals, which can serve as a foundation for further recommendations.

## Conclusion

Errors and near errors in patient identification still occur despite the use of various patient identification systems (PIS). Therefore, further improvement of PI is mandatory and might be supported by implementation of electronic PIS (ePIS). Expectations and experiences with different ePIS differ between users and those without experience in use of electronic systems. Further evaluations should include questions on how to integrate ePIS and organizational PIS in local processes in an optimized manner, how to educate professionals, and how to involve patients as active participants in their own safety environment.

### Supplementary Information

Original questions of the survey on patient identification systems
