# Supplementary material for: Usability and usefulness of (electronic) patient identification systems—A cross-sectional evaluation in German-speaking radiation oncology departments
Source: Strahlenther Onkol. 2023 Sep 15;200(6):468–74. doi: 10.1007/s00066-023-02148-9 (PMC11111529; doi:10.1007/s00066-023-02148-9)
Supplement: Supplementary file 1 — Original questions of the survey on patient identification systems [file 66_2023_2148_MOESM1_ESM.pdf]

Identifikationshilfen in der Strahlentherapie – Spielerei oder Lebensretter?

Liebe Kolleginnen und Kollegen,

herzlichen Dank für die Teilnahme an dieser Umfrage. Es handelt sich um eine Befragung der AG Patientensicherheit der DEGRO.

Ziel der Befragung ist die Erhebung gängiger Prozesse, Maßnahmen und Hilfsmittel zur Identifikation von Patientinnen und Patienten vor Behandlung.

Mit dem Absenden der Umfrage erklären Sie sich mit der Bearbeitung und Veröffentlichung einverstanden. Bitte beachten Sie die Hinweise zum Datenschutz.

Hinweis: Aus Gründen der besseren Lesbarkeit wird auf die gleichzeitige Verwendung der Sprachformen männlich, weiblich und divers (m/w/d) verzichtet. Sämtliche Personenbezeichnungen gelten gleichermaßen für alle Geschlechter.

Vielen Dank für die Teilnahme!

Diese Umfrage enthält 38 Fragen.

Demografische Informationen

[]Ich nehme zur Kenntnis, dass die Daten und Ergebnisse dieser Umfrage zu wissenschaftlichen Zwecken weiterwendet und veröffentlicht werden. *

Bitte wählen Sie eine der folgenden Antworten:

Bitte wählen Sie nur eine der folgenden Antworten aus:

- ○ Ja

[]Welcher Versorgungform sind Sie angehörig? (Mehrfachennung möglich) *

Bitte wählen Sie einen oder mehrere Punkte aus der Liste aus.

Bitte wählen Sie alle zutreffenden Antworten aus:

- ☐ Einzelpraxis
- ☐ Gemeinschaftspraxis
- ☐ Medizinisches Versorgungszentrum
- ☐ Krankenhaus der Grund- oder Regelversorgung (bis ca. 700 Betten)
- ☐ Krankenhaus der Maximalversorgung
- ☐ Universitätsklinikum

☐ Sonstiges: [ ]

[]Welcher Berufgruppe sind Sie zugeordnet? *

Bitte wählen Sie eine der folgenden Antworten:

Bitte wählen Sie nur eine der folgenden Antworten aus:

- ○ Medizinphysik
- ○ Arzt/Ärztin
- ○ MTRA
- ○ Administration
- ○ Sonstiges [ ]

[]Wie viele Patienten pro Jahr behandeln Sie in der Strahlentherapie im Schnitt? *

Bitte wählen Sie eine der folgenden Antworten:

Bitte wählen Sie nur eine der folgenden Antworten aus:

- ○ <500/Jahr
- ○ bis 1000/Jahr
- ○ bis 2500/Jahr
- ○ > 2500/Jahr
- ○ Zahlen unbekannt
- ○ Sonstiges [ ]

[]In welchem Bundesland arbeiten Sie? (optionale Angabe)

Bitte wählen Sie eine der folgenden Antworten:

Bitte wählen Sie nur eine der folgenden Antworten aus:

- ○ keine Angabe
- ○ Bayern
- ○ Baden-Württemberg
- ○ Berlin
- ○ Brandenburg
- ○ Bremen
- ○ Hamburg
- ○ Hessen
- ○ Mecklenburg-Vorpommern
- ○ Niedersachsen
- ○ Nordrhein-Westfalen
- ○ Saarland
- ○ Sachsen
- ○ Sachsen-Anhalt

- Schleswig-Holstein
- Rheinland-Pfalz
- Thüringen
- Österreich
- Schweiz

[]Haben Sie ein Konzept (organisatorisch und/oder elektronisch), mit dem Sie die Patietenidentifikation gewährleisten? *

Bitte wählen Sie eine der folgenden Antworten:

Bitte wählen Sie nur eine der folgenden Antworten aus:

- Ja
- Nein

[]
Welche Methode nutzen Sie, um Patienten vor der Bestrahlung zu identifizieren?

Sollten Sie an verschiedenen Geräten unterschiedliche Methoden nutzen, beziehen Sie sich bitte für alle weiteren Fragen auf die Methode/das Gerät mit dem höchsten Patientenanteil. Bitte schreiben Sie zusätzlich eine kurze Notiz in den Freitext am Ende dieser Frage.

*

Beantworten Sie diese Frage nur, wenn folgende Bedingungen erfüllt sind:
Antwort war 'Ja' bei Frage '6 [D05]' (Haben Sie ein Konzept (organisatorisch und/oder elektronisch), mit dem Sie die Patietenidentifikation gewährleisten?)

Bitte wählen Sie eine der folgenden Antworten:

Bitte wählen Sie nur eine der folgenden Antworten aus:

- Organisatorische Abläufe (Aufruf, Ansprache, Bildabgleich, …)
- Elektronische manuelle Abläufe (Barcode-Scanner, RFID-Chip, …)
- Elektronische automatisierte Abläufe (Finger-Scanner, Gesichtserkennung, …)
- Organisatorische und elektronische Abläufe

Bitte schreiben Sie einen Kommentar zu Ihrer Auswahl

[]
Ist die Art wie Sie Ihre Patienten identifizieren in einer Dienstanweisung/SOP festgehalten?

*

Beantworten Sie diese Frage nur, wenn folgende Bedingungen erfüllt sind:
Antwort war 'Ja' bei Frage '6 [D05]' (Haben Sie ein Konzept (organisatorisch und/oder elektronisch), mit dem Sie die Patietenidentifikation gewährleisten?)

Bitte wählen Sie eine der folgenden Antworten:

Bitte wählen Sie nur eine der folgenden Antworten aus:

- Ja
- Nein
- Nicht bekannt

Organisatorische Maßnahmen zur Identifikation

[]Welche Optionen zur Patientenidentifikation nutzen Sie? Bitte geben Sie ALLE Maßnahmen an, die im Prozess genutzt werden. *

Bitte wählen Sie einen oder mehrere Punkte aus der Liste aus.

Bitte wählen Sie alle zutreffenden Antworten aus:

- ☐ Aufruf des Namens des wartenden Patienten
- ☐ Rück-Bestätigung des Namens durch Patienten
- ☐ Abfrage des Namens des Patienten (z.B. "Bitte nennen Sie Ihren Namen")
- ☐ Abfrage des Geburtsdatums des Patienten (z.B. "Bitte nennen Sie Ihr Geburtsdatum")
- ☐ Abfrage der Adresse des Patienten (z.B. "Bitte nennen Sie Ihre Adresse")
- ☐ Abgleich des Patientenfotos
- ☐ Kontrolle eines Ausweisdokumentes
- ☐ Akten- oder Checklisten-Eintrag über erfolgte Abfrage
- ☐ Identifikation im 2-Augen-Prinzip (Abfrage durch eine Person)
- ☐ Identifikation im 4-Augen-Prinzip (Abfrage durch mindestens zwei Personen)
- ☐ Sonstiges: ____________

[]
Bei Nutzung einer <u>elektronischen</u> Dokumentation oder Checkliste: Wie wird die korrekte Identifikation im elektronischen Ablauf weiterverwendet?
*

Bitte wählen Sie eine der folgenden Antworten:

Bitte wählen Sie nur eine der folgenden Antworten aus:

- ○ Wird nicht genutzt
- ○ Identifikation wird lediglich dokumentiert
- ○ Identifikation wird im weiteren Verlauf notwendig (z.B. zur Freigabe der Türöffnung)
- ○ nicht bekannt

[]
Wie gehen Sie vor, wenn der Patient nicht auf Ihre Abfrage antworten kann? (z.B. Einschränkungen der Sprachbildung)
*

Bitte wählen Sie eine der folgenden Antworten:

Bitte wählen Sie nur eine der folgenden Antworten aus:

- ○ Abgleich mit Patientenfoto (*1 Person/2-Augen-Prinzip*)

- ◯ Abgleich mit Patientenfoto (*2 Personen/4-Augen-Prinzip*)
- ◯ Abgleich mit Ausweisdokument (*1 Person/2-Augen-Prinzip*)
- ◯ Abgleich mit Ausweisdokument (*2 Personen/4-Augen-Prinzip*)
- ◯ Abgleich mit mitgebrachten Krankenunterlagen
- ◯ Identifikation durch Begleitperson (z.B. Betreuer, Dolmetscher..)
- ◯ Bestrahlung kann nicht stattfinden
- ◯ Sonstiges [ ]

[ ]Wie regelmäßig wird die Patientenidentifikation durchgeführt? *

Bitte wählen Sie eine der folgenden Antworten:

Bitte wählen Sie nur eine der folgenden Antworten aus:

- ◯ Vor jeder Bestrahlung
- ◯ Nach einem relevantem Anteil von Terminen (z.B. ca. 10 Terminen) ist der Patient allen Mitarbeitenden bekannt und es wird <u>auf eine Identifikationsmethode verzichtet</u> (elektronisches System wird falls nötig manuell überschrieben)
- ◯ Nach einem geringem Anteil von Terminen (z.B. ca. 3 Terminen) ist der Patient allen Mitarbeitenden bekannt und es wird <u>auf eine Identifikationsmethode verzichtet</u> (elektronisches System wird falls nötig manuell überschrieben)
- ◯ Beim Wechsel des Behandlungsteams
- ◯ Nur zur Neueinstellung/Boosteinstellung
- ◯ Sonstiges [ ]

[]

Wird oder wurde in Ihrer Einrichtung über die Einführung eines elektronischen Systems zur Patientenidentifikation diskutiert?

*

Bitte wählen Sie eine der folgenden Antworten:

Bitte wählen Sie nur eine der folgenden Antworten aus:

- ◯ Ja
- ◯ Nein
- ◯ nicht bekannt

[]

Falls eine Diskussion stattgefunden hat, was waren die wesentlichen Argumente dafür oder dagegen?

*

Beantworten Sie diese Frage nur, wenn folgende Bedingungen erfüllt sind:
Antwort war 'Ja' bei Frage '13 [D07]' (Wird oder wurde in Ihrer Einrichtung über die Einführung eines elektronischen Systems zur Patientenidentifikation diskutiert?)

Bitte wählen Sie einen oder mehrere Punkte aus der Liste aus.

Bitte wählen Sie alle zutreffenden Antworten aus:

- ☐ **Dafür:** Erhöhung der Sicherheit
- ☐ **Dafür:** Nachvollziehbarkeit für Patienten
- ☐ **Dafür:** Vereinfachung der Prozesse
- ☐ **Dagegen:** Hohe Kosten
- ☐ **Dagegen:** Eingeschränkte Funktionsfähigkeit
- ☐ **Dagegen:** Hoher Aufwand für die Implementierung
- ☐ Sonstiges: ________

Elektronische Maßnahmen zur Identifikation

[]
Welche elektronischen Identifikationsinstrumente nutzen Sie? (Mehrfachantwort)

*

Kommentieren wenn eine Antwort gewählt wird

Bitte wählen Sie die zutreffenden Punkte aus und schreiben Sie einen Kommentar dazu:

- [ ] Barcode (z.B. Armband)
- [ ] ID-Karte (z.B. mit RFID-Chip)
- [ ] Fingerabdrucksensor
- [ ] Gesichtserkennung
- [ ] Körpererkennung (Lagerung)

Sonstiges:

[]
Seit wann nutzen Sie ein elektronisches Identifikations-System?

*

Bitte wählen Sie eine der folgenden Antworten:

Bitte wählen Sie nur eine der folgenden Antworten aus:

- ◯ Seit weniger als 1 Jahr
- ◯ Seit 1 - 3 Jahren
- ◯ Seit mehr als 3 Jahren
- ◯ nicht bekannt

[]Welches elektronische Identifikationssystem (bzw. welchen Hersteller) nutzen Sie? (Optional)

Kommentieren wenn eine Antwort gewählt wird

Bitte wählen Sie die zutreffenden Punkte aus und schreiben Sie einen Kommentar dazu:

- [ ] Opasca (Patientenvalidierung)
- [ ] C-Rad (cPatient/cAutoVerify)

☐ VisionRT (SafeID)

☐ Elekta (Tungsten)

☐ Siemens

☐ mediloX (Patientenarmband)

☐ Wolf und Appenzeller (Patientenarmband)

Sonstiges:

[]
Wie häufig kommt es vor, dass das elektronische Identifikations-System wiederholt angewendet werden muss, da die Identifikation fehlgeschlagen ist?

Bitte wählen Sie die Antwortoption aus, welche am ehesten zutrifft.

*

Bitte wählen Sie eine der folgenden Antworten:

Bitte wählen Sie nur eine der folgenden Antworten aus:

- ○ nie
- ○ Etwa 1 x pro Quartal
- ○ Etwa 1 x pro Monat
- ○ Etwa 1 x pro Woche
- ○ Täglich
- ○ Häufigkeit unbekannt
- ○ Sonstiges

[]
Wie häufig kommt es vor, dass das elektronische Identifikations-System schlussendlich keine Patientenidentifikation ermöglicht?

Bitte wählen Sie die Antwortauswahl, welche am ehesten zutrifft.

*

Bitte wählen Sie eine der folgenden Antworten:

Bitte wählen Sie nur eine der folgenden Antworten aus:

- nie
- Etwa 1 x pro Quartal
- Etwa 1 x pro Monat
- Etwa 1 x pro Woche
- Täglich
- Häufigkeit unbekannt
- Sonstiges

[]
Wie gehen Sie vor, wenn das elektronische Identifikations-System schlussendlich nicht funktioniert („Time-Out")?
*

Bitte wählen Sie eine der folgenden Antworten:

Bitte wählen Sie nur eine der folgenden Antworten aus:

- Behandlung kann nicht angewendet werden, solange keine erfolgreiche elektronische Identifikation stattgefunden hat
- Identifikation wird durch *1 Person/2-Augen-Prinzip* anhand der Abfrage Name / Geburtstag / Adresse durchgeführt und die elektronische Abfrage wird überschrieben
- Identifikation wird durch *2 Personen/4-Augen-Prinzip* anhand der Abfrage Name / Geburtstag / Adresse durchgeführt und die elektronische Abfrage wird überschrieben
- nicht bekannt
- Sonstiges

[]Würden Sie Ihr elektronisches Identifikations-Systems weiterempfehlen? *

Bitte wählen Sie einen oder mehrere Punkte aus der Liste aus.

Bitte wählen Sie alle zutreffenden Antworten aus:

- ja
- nein, zu fehleranfällig
- nein, zu aufwändig
- keine Aussage
- Sonstiges:

[]
Wie regelmäßig wird die elektronische Patientenidentifikation durchgeführt? Bitte wählen Sie die Antwortoption aus, die am ehesten zutrifft.
*

Bitte wählen Sie eine der folgenden Antworten:

Bitte wählen Sie nur eine der folgenden Antworten aus:

- ○ Vor jeder Bestrahlung
- ○ Nach einem relevanten Anteil von Terminen (Z.b. ca. 10 Terminen) ist der Patient allen Mitarbeitenden bekannt und es wird auf eine Identifikationsmethode verzichtet (elektronisches System wird falls nötig manuell überschrieben)
- ○ Nach einem geringen Anteil von Terminen (Z.b. ca. 3 Terminen) ist der Patient allen Mitarbeitenden bekannt und es wird auf eine Identifikationsmethode verzichtet (elektronisches System wird falls nötig manuell überschrieben)
- ○ Beim Wechsel des Behandlungsteams
- ○ Nur zur Neueinstellung/Boosteinstellung
- ○ nicht bekannt
- ○ Sonstiges [ ]

Probleme mit der Patientenidentfikation

[]
Ereignete sich in Ihrer Abteilung augrund einer Patientenverwechslung ein beinahe-Ereignis (ein Fehler, der nicht zu einer Fehlbestrahlung geführt hat/nicht meldepflichtiges Vorkommnis) in den letzten 5 Jahren? (Bitte beachten Sie den Hinweis

*

Bitte wählen Sie eine der folgenden Antworten:

Bitte wählen Sie nur eine der folgenden Antworten aus:

- nein
- Ja, Anzahl nicht bekannt
- Ja, weniger als 5
- Ja, mehr als 5
- keine Aussage
- Sonstiges

Hinweis: analog StrlSchV Anlage 14 VII gilt als meldepflichtig auch ein beinahe-Ereignis, welches außerhalb der qualitätssichernden Maßnahmen entdeckt wurde

[]Im Rahmen welches Prozessschrittes kam es am häufigsten zur Entdeckung eines beinahe-Ereignis im Sinne einer Patientenverwechslung? *

Beantworten Sie diese Frage nur, wenn folgende Bedingungen erfüllt sind:
Antwort war NICHT 'nein' bei Frage '23 [P01]' (Ereignete sich in Ihrer Abteilung augrund einer Patientenverwechslung ein beinahe-Ereignis (ein Fehler, der nicht zu einer Fehlbestrahlung geführt hat/nicht meldepflichtiges Vorkommnis) in den letzten 5 Jahren? (Bitte beachten Sie den Hinweis)

Bitte wählen Sie eine der folgenden Antworten:

Bitte wählen Sie nur eine der folgenden Antworten aus:

- Ankunft/Anmeldung des Patienten in der Abteilung
- Informationsabfrage durch Personal am Bestrahlungsgerät
- Lagerung
- Verifikationsaufnahme
- keine Aussage
- Sonstiges

[]
Im Rahmen welches Prozessschrittes ereigneten sich die Mehrzahl der beinahe-Ereignisse im Sinne einer Patientenverwechslung ?

*

Beantworten Sie diese Frage nur, wenn folgende Bedingungen erfüllt sind:
Antwort war NICHT 'nein' bei Frage '23 [P01]' (Ereignete sich in Ihrer Abteilung augrund einer Patientenverwechslung ein beinahe-Ereignis (ein Fehler, der nicht zu einer Fehlbestrahlung geführt hat/nicht meldepflichtiges Vorkommnis) in den letzten 5 Jahren? (Bitte beachten Sie den Hinweis)

Bitte wählen Sie eine der folgenden Antworten:

Bitte wählen Sie nur eine der folgenden Antworten aus:

- Ankunft/Anmeldung des Patienten in der Abteilung
- Informationsabfrage durch Personal am Bestrahlungsgerät
- Lagerung
- Verifikationsaufnahme
- keine Aussage
- Sonstiges

[]Welcher Faktor war nach Ihrer Einschätzung hauptverantwortlich für die Mehrzahl der beinahe-Ereignisse im Sinne einer Patientenverwechslung? *

Beantworten Sie diese Frage nur, wenn folgende Bedingungen erfüllt sind:
Antwort war NICHT 'nein' bei Frage '23 [P01]' (Ereignete sich in Ihrer Abteilung augrund einer Patientenverwechslung ein beinahe-Ereignis (ein Fehler, der nicht zu einer Fehlbestrahlung geführt hat/nicht meldepflichtiges Vorkommnis) in den letzten 5 Jahren? (Bitte beachten Sie den Hinweis)

Bitte wählen Sie eine der folgenden Antworten:

Bitte wählen Sie nur eine der folgenden Antworten aus:

- Menschliche Faktoren
- Software-Faktoren
- Hardware-Faktoren
- keine Aussage
- Sonstiges

[]
Ereignete sich in Ihrer Abteilung augrund einer Patientenverwechslung ein nach StrlSchV meldepflichtiges Vorkommnis in den letzten 5 Jahren?

*

Bitte wählen Sie eine der folgenden Antworten:

Bitte wählen Sie nur eine der folgenden Antworten aus:

- nein
- Ja, Anzahl nicht bekannt
- Ja, weniger als 5
- Ja, mehr als 5

- keine Aussage
- Sonstiges

[]Im Rahmen welches Prozessschrittes ereigneten sich die Mehrzahl der Vorkommnisse? *

Beantworten Sie diese Frage nur, wenn folgende Bedingungen erfüllt sind:
Antwort war NICHT 'nein' bei Frage '27 [P05]' (Ereignete sich in Ihrer Abteilung augrund einer Patientenverwechslung ein nach StrlSchV meldepflichtiges Vorkommnis in den letzten 5 Jahren?)

Bitte wählen Sie eine der folgenden Antworten:

Bitte wählen Sie nur eine der folgenden Antworten aus:

- Ankunft/Anmeldung des Patienten in der Abteilung
- Informationsabfrage durch Personal am Bestrahlungsgerät
- Lagerung
- Verifikationsaufnahme
- keine Aussage
- Sonstiges

[]
Welcher Faktor war nach Ihrer Einschätzung hauptverantwortlich für die Mehrzahl der Vorkommnisse?
*

Beantworten Sie diese Frage nur, wenn folgende Bedingungen erfüllt sind:
Antwort war NICHT 'nein' bei Frage '27 [P05]' (Ereignete sich in Ihrer Abteilung augrund einer Patientenverwechslung ein nach StrlSchV meldepflichtiges Vorkommnis in den letzten 5 Jahren?)

Bitte wählen Sie eine der folgenden Antworten:

Bitte wählen Sie nur eine der folgenden Antworten aus:

- Menschliche Faktoren
- Software-Faktoren
- Hardware-Faktoren
- keine Aussage
- Sonstiges

[]Denken Sie, dass ein elektronisches System die Mehrzahl der beinahe-Ereignisse bzw. Vorkommnisse hätte verhindern können? *

Bitte wählen Sie eine der folgenden Antworten:

Bitte wählen Sie nur eine der folgenden Antworten aus:

- Ja
- nein

- ◯ Keine Aussage

Erfahrungen mit Identifikationssystemen

Nachfolgend werden verschiedene Meinungen aufgeführt. Sie können sich anhand der Skala bei jeder Meinung entscheiden, ob sie ihr:

deutlich widersprechen, **eher widersprechen**, **sie neutral bewerten, eher zustimmen,** oder **deutlich zustimmen.**

[]Die Aufmerksamkeit des Personals lässt mit der Nutzung eines elektronischen Identifikations-Systems nach. *

Bitte wählen Sie eine der folgenden Antworten:

Bitte wählen Sie nur eine der folgenden Antworten aus:

- widerspreche deutlich
- widerspreche
- neutral
- stimme zu
- stimme deutlich zu

[]
Das Personal fühlt sich sicherer mit der Nutzung eines rein organisatorischen Systems zur Identifizierung der Patienten

*

Bitte wählen Sie eine der folgenden Antworten:

Bitte wählen Sie nur eine der folgenden Antworten aus:

- widerspreche deutlich
- widerspreche
- neutral
- stimme zu
- stimme deutlich zu

[]
Die Patienten fühlen sich sicherer mit der Nutzung eines elektronischen Identifikations-Systems.

*

Bitte wählen Sie eine der folgenden Antworten:

Bitte wählen Sie nur eine der folgenden Antworten aus:

- widerspreche deutlich
- widerspreche
- neutral
- stimme zu
- stimme deutlich zu

[]Ein elektronisches System entlastet das Personal und es kann sich auf andere Aufgaben konzentrieren. *

Bitte wählen Sie eine der folgenden Antworten:

Bitte wählen Sie nur eine der folgenden Antworten aus:

- widerspreche deutlich
- widerspreche
- neutral
- stimme zu
- stimme deutlich zu

[]Ein organisatorisches System zur Identifizierung der Patienten bietet einen sehr hohen Sicherheitsstandard. *

Bitte wählen Sie eine der folgenden Antworten:

Bitte wählen Sie nur eine der folgenden Antworten aus:

- widerspreche deutlich
- widerspreche
- neutral
- stimme zu
- stimme deutlich zu

[]
Der zusätzliche Sicherheitsaspekt rechtfertigt sowohl die Kosten als auch den erhöhten Aufwand eines elektronischen Identifikations-Systems (Kosten/Nutzen-Faktor).

*

Bitte wählen Sie eine der folgenden Antworten:

Bitte wählen Sie nur eine der folgenden Antworten aus:

- widerspreche deutlich
- widerspreche
- neutral
- stimme zu
- stimme deutlich zu

[]
Trotz der Nutzung eines elektronischen Systems sollte ein organisatorisches System ergänzt sein.

*

Bitte wählen Sie eine der folgenden Antworten:

Bitte wählen Sie nur eine der folgenden Antworten aus:

- widerspreche deutlich
- widerspreche
- neutral
- stimme zu
- stimme deutlich zu

[]Bitte wählen (optional) Sie eine 5-stellige Zufallszahl, die von allen Mitarbeitern Ihrer Abteilung genutzt wird, die diese Umfrage ausfüllen (z.B. 47809, bitte wählen Sie nicht 12345) . So können die Fragebögen korreliert werden, ohne die Anonymität der Teilnehmenden aufzuheben. Vielen Dank!

Bitte geben Sie Ihre Antwort hier ein:

Wir danken Ihnen für die Teilnahme.
08.11.2022 – 10:16

Übermittlung Ihres ausgefüllten Fragebogens:
Vielen Dank für die Beantwortung des Fragebogens.
